# Emerging Artemisinin Partial Resistance in Southern Africa

**DOI:** 10.4269/ajtmh.25-0417

**Published:** 2026-03-17

**Authors:** Rosario Martinez-Vega, Deus S. Ishengoma, Roly Gosling

**Affiliations:** ^1^ Department of Disease Control, London School of Hygiene and Tropical Medicine, London, United Kingdom;; ^2^ National Institute for Medical Research, Dar es Salaam, Tanzania;; ^3^ Ifakara Health Institute, Dar es Salaam, Tanzania;; ^4^ Malaria Elimination Initiative, Institute of Global Health Sciences, University of California San Francisco, San Francisco, California, USA

## Abstract

A new focus of probable artemisinin-partial resistance (ART-R) has been identified in southern Africa, driven by the P441L mutation in the *Plasmodium falciparum* Kelch13 gene. This emerging cross-border cluster across Angola, Namibia, and Zambia represents the fourth major ART-R focus identified in Africa, adding to the previously reported foci in East Africa and the Horn of Africa. Findings from three new studies reveal 7.3%, 33%, and 47% prevalence of P441L in specific sites in Angola, Namibia, and Zambia, respectively. The Genomics of Malaria in the Elimination 8 project enabled harmonized, real-time molecular surveillance across five countries, providing actionable data for national malaria programs. Given past failures to contain chloroquine and sulfadoxine-pyrimethamine resistance, a timely response is critical. We recommend immediate treatment policy shifts, expanded surveillance, and regional coordination to prevent the spread of ART-R. This Perspective calls on global health actors to act decisively while resistance remains geographically limited.

## INTRODUCTION

In this Perspective, we aim to bring to the attention of global health security and global health experts the findings of three papers published in the *American Journal of Tropical Medicine and Hygiene* describing a new focus of probable artemisinin-partial resistance (ART-R) in southern Africa, driven by the P441L mutation in the *Plasmodium falciparum* kelch13 (*Pfk13*) gene.^1^^–^^3^ Identified through a regional surveillance and capacity-building initiative, this cross-border cluster spans Angola, Namibia, and Zambia, representing the fourth ART-R focus identified in Africa, following three independently emerged foci: one centered in Rwanda, with spread into Tanzania and Uganda, characterized by the 561H mutation; a northern Uganda cluster marked by the C469Y and A675V mutations; and a Horn of Africa cluster in Eritrea and Ethiopia associated with the R622I mutation. Notably, the P441L mutation has also been detected at various prevalences in these regions. Molecular surveillance in southern Africa was designed across five countries with the aim of providing geographically representative data, rapidly ready for country and regional decision-making. Harmonized sampling, data collection, and analysis methodologies enabled cross-country comparisons. The early detection and limited geographical spread of this cluster (Figure 1) highlight the need for immediate actions by National Malaria Programs (NMPs) to curb the spread while ART-R mutations remain localized.

**Figure 1.**
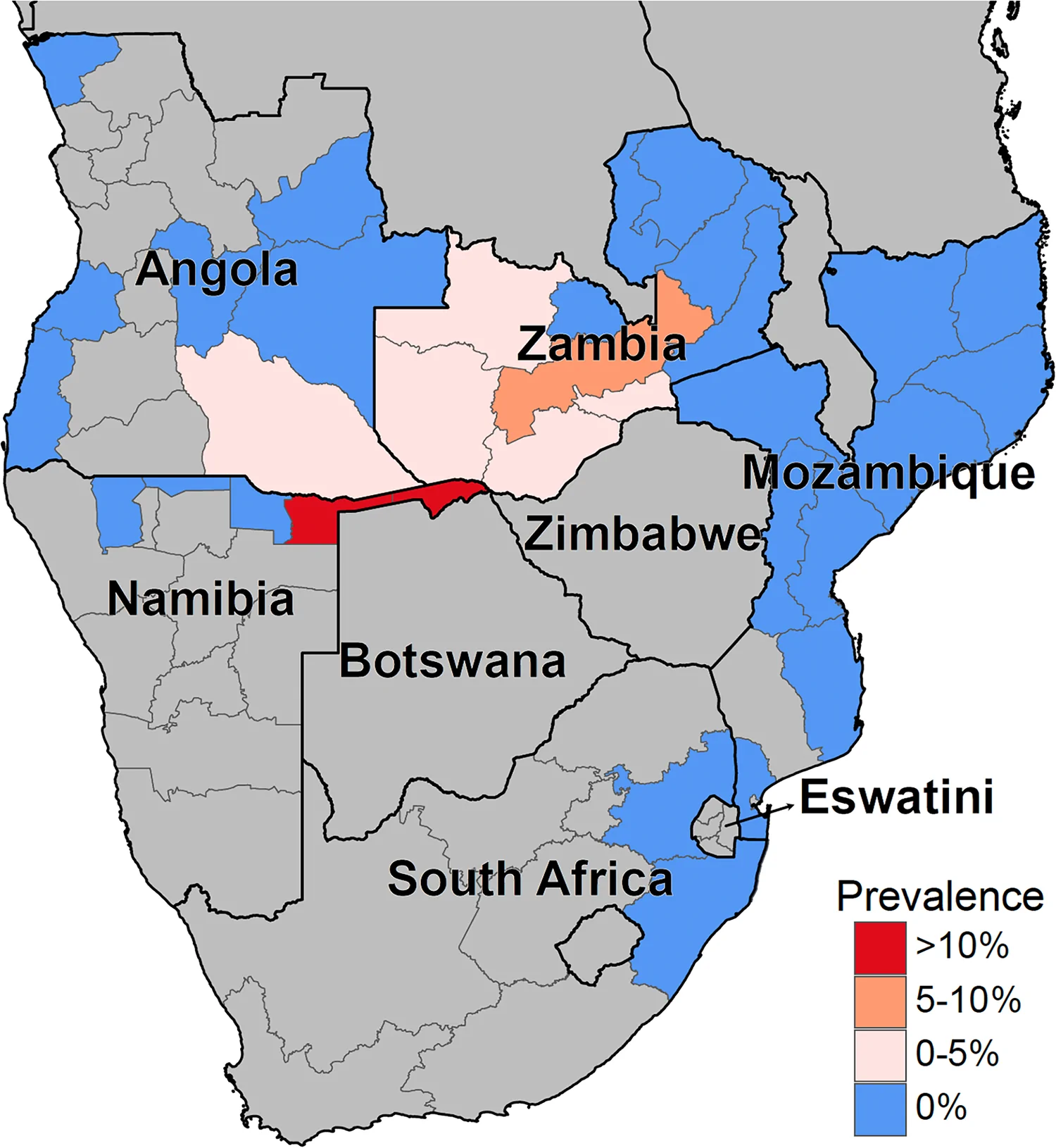
Sub-national prevalence of the *PfK13* P441L mutation across southern Africa (courtesy of GenE8 and GenMoz Projects).

### Drug resistance and the cross-border subregional challenge.

Delayed recognition of chloroquine and sulfadoxine-pyrimethamine resistance in the late 1990s and early 2000s taught us major lessons, with backtracking of malaria control efforts, significant costs, and preventable loss of life.^4^^–^^6^ Molecular surveillance is now widely used in malaria endemic countries and enables early detection of emerging resistance.^7^ Within the last decade, *Pfk13* mutations have emerged and spread within and across African countries.^8^ However, not all *Pfk13* mutations are associated with ART-R. The World Health Organization (WHO) classifies mutations as “validated” when supported by both clinical and laboratory evidence of delayed parasite clearance, while “candidate” mutations have emerged, but with inconclusive evidence for delayed clearance.^9^ To date, WHO has validated thirteen mutations in the *Pfk13* propeller domain and identified nine additional candidate mutations, including P441L. Validated mutations in Africa include R561H in Rwanda, Tanzania, and Uganda; C469Y and A675V in northern Uganda; and R622I in Eritrea and Ethiopia.^10^ Policy discussions are underway in East Africa, with plans to pilot first-line antimalarial drug switches in development, though implementation is pending due to financing constraints. In the Horn of Africa, these conversations are only just beginning, with few mitigation measures planned so far.

The relationship between *Pfk13* mutations and definitive ART-R is complex, likely involving additional unknown mutations or mechanisms.^11^^,^^12^ Definitive resistance confirmation requires therapeutic efficacy studies (TES) examining parasitemia on day 3 after therapy or parasite clearance rates, genomic analyses, and specific *in vivo* or *ex vivo* laboratory assays such as ring-stage survival assays.^13^ Although such confirmation is pending for the southern African cluster, the emergence of P441L in established ART-R hotspots in Uganda, Rwanda, and Tanzania makes TES implementation in affected areas an urgent priority.

### GenE8 Project findings: Angola, Namibia, and Zambia.

The Genomics of Malaria in the Elimination 8 (GenE8) project aims to build malaria molecular surveillance capacity and generate actionable data in southern African countries targeting malaria elimination. This initiative aligns with the goals of the Southern African Development Community’s Elimination 8, a regional partnership comprising Angola, Botswana, Eswatini, Mozambique, Namibia, South Africa, Zambia, and Zimbabwe. While the E8 includes eight countries, only five participated in GenE8, with Mozambique, Botswana, and Zimbabwe not taking part. The three newly published papers represent early outputs of GenE8 and provide a snapshot of likely ART-R emergence in southern Africa in 2023.^1^^–^^3^ The data suggest that ART-R is gaining a foothold and threatening the efficacy of frontline therapies.

In Angola, where genotype-corrected failure of artemether-lumefantrine (AL) in over 10% of study subjects has been previously reported in some parts of the country,^14^ the recent discovery of P441L in the southeast region represents a potentially important signal, although its clinical relevance remains unconfirmed. Genomic surveillance across eight provinces revealed geographic heterogeneity in resistance markers, with emerging signals of ART-R in the southeast. The P441L candidate ART-R marker was detected in 7.3% of samples from Calai municipality, bordering Namibia, while the validated P574L ART-R marker was found in one sample from Moxico province.^15^

In Namibia, a country with overall low malaria transmission approaching elimination, genomic data revealed a surprisingly high prevalence of validated or candidate *PfK13* ART-R mutations in treated cases. A cross-sectional survey in five northern regions, where malaria transmission remains comparatively high, revealed a 33% prevalence of the candidate P441L ART-R marker, most prominently in the Zambezi region. Additionally, validated P574L and A675V ART-R markers were detected at low levels. These findings raise concerns about sustained local transmission of ART-R in a country nearing malaria elimination.^2^

In Zambia, genomic surveillance across 9 districts and 5 provinces found P441L in 3.1% of sequenced samples. When extrapolated to the national level, the estimated prevalence was 1.4%. Prevalence of the mutation peaked at 47% in a single site in the Central Province, where other mutations such as P574L were also identified. Notably, P441L was not observed in Zambia’s northern provinces, where malaria transmission is highest, but rather in the western and central parts of the country, which are characterized by lower transmission intensity. These data highlight the threat posed by ART-R in low-transmission settings, where therapeutic monitoring has been historically limited.^1^ These findings align with those from neighboring Angola and Namibia, providing a compelling suggestion of cross-border circulation of ART-R parasites in southern Africa.

Across all three countries, the *mdr1* N86 genotype, known to be associated with reduced susceptibility to lumefantrine, was found at near-fixation levels (>90%), underscoring an additional threat to AL efficacy.^16^

### Why Southern AFRICA?

While the origin of the *Pfk13* P441L mutation remains unclear, its detection across multiple sites in southern Africa warrants consideration of the ecological and programmatic drivers of emergence and spread. Relatively low malaria transmission in this region, a pattern also observed in most areas where ART-R has become established, such as Rwanda, Eritrea, Ethiopia, and the Greater Mekong Subregion, may favor the survival of resistant strains due to reduced parasite recombination. Other contributing factors include first, consistent drug pressure from AL use across all countries, favoring resistance selection, second, significant cross-border migration, particularly among agricultural workers with limited access to quality health care and exposure to substandard treatments, and third, limited TESs in low transmission areas, impeding timely detection and response.

### Next steps: Urgent regional action required.

While the extent of the problem in southern Africa is still not fully defined, urgent and definitive actions should be taken to mitigate the spread of ART-R in the region.

First, TESs that measure both parasite clearance rates and prevalence of *Pfk13* mutations should be initiated in Angola, Namibia, and Zambia, where P441L prevalence is highest. Designs should reflect local contexts, including both cross-border recruitment and active and passive case detection. Innovative study designs such as the parasite clearance and prevention of infection (PCPI) studies^17^^,^^18^ may provide additional insights into the clinical relevance of the P441L mutation. The PCPI model is a pragmatic, randomized controlled trial framework designed to evaluate both the efficacy of antimalarial treatments and the ability of different parasite genotypes to survive and transmit following treatment. It combines close monitoring of parasite clearance with follow-up to detect new infections, allowing assessment of both treatment performance and transmission potential. Incorporating such approaches could enhance our understanding of ART-R dynamics and guide more targeted interventions.

Second, expanded geographical surveillance is needed to better define the distribution and potential spread of the P441L mutation. This requires finer-scale mapping of parasites using intensive sampling in areas where P441L has already been detected (southeast Angola, western and central Zambia) and extending to Botswana and Zimbabwe to assess whether the P441L mutation has emerged or spread across country borders. We recommend repeating subregional sampling every two years to monitor and evaluate intervention effectiveness.

Third, NMPs should be supported to switch first-line treatment away from AL in areas threatened by potentially drug-resistant strains. The switching needs to be part of country ART-R mitigation plans and may involve a straightforward switch or be incorporated into a multiple first-line therapy (MFT) or antimalarial drug rotation strategy. Such strategies aim to preserve the efficacy of artemisinin by protecting its partner drugs, which are often the first to be compromised when resistance emerges. Loss of partner drug efficacy can lead to suboptimal treatment, placing selective pressure on the artemisinin component, and accelerating resistance. By rotating or combining therapies, especially through the introduction of alternative ACTs or triple ACTs,^19^ where two partner drugs accompany the artemisinin component, programs can reduce this selective pressure and delay resistance development. NMPs may benefit from market-shaping financing to support the introduction of alternative ACTs, triple ACTs,^19^ or new non-artemisinin antimalarial combination drugs when available.

Fourth, strengthening access to high-quality diagnosis and treatment at the community level is essential, especially for hard-to-reach and mobile populations. The E8 previously received Global Fund support to reach mobile migrant populations, but that funding has since ended, leaving a critical gap in service delivery for highly mobile groups in this area.

Finally, we must optimize vector control by ensuring access to and effective use of proven interventions, particularly indoor residual spraying, insecticide-treated nets, or other tools using insecticides with confirmed efficacy.

## CONCLUSION

The identification of a southern African cluster of potential ART-R *P. falciparum* parasites reflects the foresight of the E8 initiative and its partners in establishing a regional molecular surveillance network. However, surveillance can only contribute to containment if the data are acted upon swiftly and strategically.

While the P441L mutation remains a candidate marker, its unexpectedly high prevalence in parts of Zambia, Angola, and Namibia, combined with the almost complete dominance of the *mdr1* N86 genotype known to reduce susceptibility to lumefantrine, raises concerns about continued reliance on AL in these settings. In the short term, countries should consider introducing alternative ACTs through coordinated cross-border strategies, while concurrently carrying out TESs focused on areas and genotypes of concern.

The challenge now lies in translating surveillance data into coordinated action in a period of reduced financing. We must foster cross-border collaboration and harness our combined energies to act before artemisinin resistance becomes widespread and entrenched in southern Africa.
